# Reviewer acknowledgement 2013

**DOI:** 10.1186/1750-1172-8-39

**Published:** 2013-03-28

**Authors:** Ségolène Aymé

**Affiliations:** 1 Orphanet - INSERM US14, Rare Disease Platform, 96 rue Didot, 75014 Paris, France

## Abstract

**Contributing reviewers:**

The editors of Orphanet Journal of Rare Diseases would like to thank all our reviewers who have contributed to the journal in volume 7 (2012).

## 

Ronald Admiraal

Netherlands

Patricia Aguilar Martinez

France

Zubair Ahmed

United States of America

S. Faisal Ahmed

United Kingdom

Jan Albrecht

Poland

Beate Albrecht

Germany

Rando Allikmets

United States of America

Margarida Amaral

Portugal

Christer Andersson

Sweden

David Araujo-Vilar

Spain

Eloisa Arbustini

Italy

Pamela Arn

United States of America

Martin Ashton-Key

United Kingdom

Patrick Aubourg

France

Michael Badminton

United Kingdom

Raffaele Badolato

Italy

David Baguley

United Kingdom

Kate Baker

United Kingdom

Ivo Barić

Croatia

Timothy Barrett

United Kingdom

Frank Bartel

United States of America

Peter Barth

Netherlands

Lina Basel-Vanagaite

Israel

Roberta Battini

Italy

Matthias Baumgartner

Switzerland

Michael Beck

United States of America

David Bedwell

United States of America

Marianna Bei

United States of America

Bruno Bembi

Italy

Michael Bennett

United States of America

Tamar Ben-Yosef

Israel

Bruria Ben-Zeev

Israel

Johannes Berger

Austria

Jean-Francois Bernaudin

France

Roberto Berni Canani

Italy

Gerard Berry

United States of America

Enrico Bertini

Italy

H. Berna Beverloo

Netherlands

Valerie Biancalana

France

Daniel Bichet

Canada

Luigi Bisceglia

Italy

Ignacio Blanco

Spain

Agnes Bloch-Zupan

France

Anna Blom

Sweden

Cornelius Boerkoel

United States of America

Chantal Boisvert

United States of America

Luisa Bonafé

Switzerland

Renato Borgatti

Italy

Salud Borrego

Spain

Dorin Bogdan Borza

United States of America

Patrice Bouvagnet

France

Olivier Braissant

Switzerland

Francesco Brancati

Italy

Maria Luisa Brandi

Italy

Attila Braun

Germany

Nancy Braverman

Canada

Sara Yvonne Brucker

Germany

Han Brunner

Netherlands

Deborah Bruns

United States of America

Catherine Caillaud

France

Christine Cans

France

Andrew Cant

United Kingdom

Wei Cao

United States of America

David Cassiman

Belgium

Patricia Celestino-Soper

United States of America

Hassen Chaabani

Tunisia

Anupam Chakrapani

United Kingdom

Lawrence Chan

United States of America

Philippe Chanson

France

Anthi Chatzikyriakidou

Greece

Chia-Yu Chu

Taiwan

Rubin Cohen

United States of America

Marina Colombi

Italy

Louise Corben

Australia

Mary Corey

Canada

Valerie Cormier-Daire

France

Mireille Cossee

France

Ulrich Costabel

Germany

Timothy Martin Cox

United Kingdom

Bruno Crestani

France

Michael Davies

Australia

Alessandra d'Azzo

United States of America

Philippe de Moerloose

Switzerland

Jacques de Mouzon

France

Anne De Paepe

Belgium

Marianne de Visser

Netherlands

Darryl De Vivo

United States of America

Claude Desnuelle

France

Robin Deterding

United States of America

Jean-Charles Deybach

France

George Diaz

United States of America

Angela Dispenzieri

United States of America

Jean Donadieu

France

Dian Donnai

United Kingdom

Dennis Drayna

United States of America

Daniel Driscoll

United States of America

Sarah Dyack

Canada

Thomas Eggermann

Germany

Tore Eid

United States of America

Deborah Elstein

Israel

Regina Ensenauer

Germany

Inigo Espinosa

Spain

Gareth Evans

United Kingdom

Paola Facchin

Italy

Marie Faughnan

Canada

François Feillet

France

Alessandra Ferlini

Italy

Leslie Findley

United Kingdom

Brian Fowler

Switzerland

Sixto Garcia-Minaur

Spain

Abhimanyu Garg

United States of America

Matthew Gentry

United States of America

Dominique Germain

France

Ruth Gershoni-Baruch

Israel

Daniele Ghezzi

Italy

Giorgio Gimelli

Italy

Maria Gizewska

Poland

Maurice Godfrey

United States of America

Cyril Goizet

France

Paola Grammatico

Italy

David Greenberg

United States of America

Danielle Grenier

Canada

Matthias Griese

Germany

Daniel Grinberg

Spain

Paola Griseri

Italy

Samir Gupta

Canada

Smail Hadj-Rabia

France

Johannes Haeberle

Switzerland

Jane Halliday

Australia

Philip Hawkins

United Kingdom

Madhuri Hegde

United States of America

Raoul Hennekam

Netherlands

Lothar Hennighausen

United States of America

Karen Herbst

United States of America

Frederik Hes

Netherlands

Richard Hift

South Africa

Robert Hirst

United Kingdom

Hal Hoffman

United States of America

Georg Hoffmann

Germany

Carla Hollak

Netherlands

Rita Horvath

United Kingdom

Juliane Hoyer

Germany

Khalid Hussain

United Kingdom

Caroline Huyard

France

Wuh-Liang Hwu

Taiwan

Ken Ishii

Japan

Anthony Isles

United Kingdom

Bruce Jacobson

United States of America

Gregory Jicha

United States of America

Colin Johnson

United Kingdom

Simon Johnson

United Kingdom

Luba Kalaydjieva

Australia

Asgar Kalla

South Africa

Christoph Kampmann

Germany

Paige Kaplan

United States of America

Chung How Kau

United States of America

Stephan Kemp

Netherlands

Babak Khoshnood

France

Farid Kianifard

United States of America

Myungshin Kim

South Korea

Alfried Kohlschütter

Germany

Haris Kokotas

Greece

Anna Kole

United States of America

Isabelle Kone Paut

France

Uwe Kornak

Germany

Christian Körner

Germany

Tomoki Kosho

Japan

Deborah Krakow

United States of America

Odile Kremp

France

Kerstin Kutsche

Germany

Anne-Marie Laberge

Canada

Stefan Lange

Germany

Giovanna Lattanzi

Italy

William Lawson

United States of America

Martine Le Merrer

France

Dirk Lefeber

Netherlands

James Leonard

United Kingdom

Michelle Letarte

Canada

Hubert Leufkens

Netherlands

Kui Liu

Sweden

Manuel Lopez

United States of America

Allan Meldgaard Lund

Denmark

James Lupski

United States of America

Tomoko Makishima

United States of America

Pineda Marfa

Spain

Branka Marinovic

Croatia

Francesco Marrosu

Italy

Jacob Mashiah

Israel

Kim McBride

United States of America

Jennifer McGinley

Australia

Divya Mehrotra

India

Eugen Mengel

Germany

Sandrine Meunier

France

Sabine Mignot

France

Daria Mochly-Rosen

United States of America

Klaus Mohnike

Ghana

Ute Moog

Germany

María-Josefa Morán-Jiménez

Spain

Ulrike Muetze

Germany

John Newton

United Kingdom

Vincenzo Nigro

Italy

Wataru Nishie

Japan

Ichizo Nishino

Japan

Alan Nurden

France

Devin Oglesbee

United States of America

Torayuki Okuyama

Japan

Osamu Onodera

Japan

Karen Helene Orstavik

Norway

Daniel Ory

United States of America

John Ostergaard

Denmark

Giovanni Pagano

Italy

Richard Paisey

United Kingdom

Giovanni Palladini

Italy

Federico Pallardo

Spain

Juan Pascual

United States of America

Gregory Pastores

United States of America

Kyle Peake

Canada

Nicoletta Pedemonte

Italy

Flora Peyvandi

Italy

Jean-Yves Picard

France

Richard Pierce

United States of America

Yves Pirson

Belgium

Venerino Poletti

Italy

Raphaël Porcher

France

Andrea Poretti

United States of America

Forbes Porter

United States of America

Manuel Posada

Spain

Markus Preising

Germany

Prem Puri

Ireland

Shamima Rahman

United Kingdom

Manoj Ramachandran

United Kingdom

Kunal Ray

India

Jennita Reefhuis

United States of America

Ernst Reichenberger

United States of America

Carlo Rivolta

Switzerland

William Rizzo

United States of America

Carlos Robalo Cordeiro

Portugal

Peter Robinson

Germany

Julia Rohayem

Germany

Arndt Rolfs

Germany

Hans-Dieter Rott

Germany

Francois Rousseau

Canada

Jean-Christophe Roux

France

Tony Rupar

Canada

S. Lane Rutledge

United States of America

Yoshiaki Saito

Japan

Lynn Sakai

United States of America

David Sarraf

United States of America

Jean-Marie Saudubray

France

Maurizio Scarpa

Italy

Gunter Scharer

United States of America

Raphael Schiffmann

United States of America

Michael Schirmer

Austria

Enno Schmidt

Germany

Susanne Schneider

Germany

Xiaoye Schneider-Yin

Switzerland

Ludger Schöls

Germany

Benedikt Schoser

Germany

Edward Schuchman

United States of America

Alfredo Scillitani

Italy

Gian Domenico Sebastiani

Italy

Frederic Sedel

France

Zdenek Sedlacek

Czech Republic

Adalberto Sessa

Italy

Lee Shapiro

United States of America

Trevor Shepherd

Canada

Eileen Shore

United States of America

Elena Sieni

Italy

Eduardo Silva

Portugal

Shweta Singh

Malaysia

Andrew Singleton

United States of America

Joseph Sisson

United States of America

Erik Snapp

United States of America

Orna Staretz-Chacham

Israel

Steven Steinberg

United States of America

Colin Steward

United Kingdom

Jan Stolk

Netherlands

Björn Stollenwerk

Germany

Arnold Strauss

United States of America

Pasquale Striano

Italy

Eduard Struys

Netherlands

Oksana Suchowersky

Canada

Masatoshi Suzuki

United States of America

Jolanta Sykut-Cegielska

Poland

Atsuhito Takeda

Japan

Stephan Tanner

United States of America

Abdellatif Tazi

France

Yuichi Teraki

Japan

Nalin Thakker

United Kingdom

Jennifer Thorne

United States of America

Nicki Tiffin

South Africa

Sanna Toiviainen-Salo

Finland

James Tonsgard

United States of America

Vicente Torres

United States of America

Toni Torresani

Switzerland

Valerie Touitou

France

Lisbeth Tranebjaerg

Denmark

Lennart Truedsson

Sweden

Sylvie Tuffery-Giraud

France

Peter Turnpenny

United Kingdom

Zsolt Urban

United States of America

Matilde Valeria Ursini

Italy

Vassili Valayannopoulos

France

Valentino Valentini

Italy

Ludo Van Den Bosch

Belgium

Ambro van Hoof

United States of America

Rick van Minkelen

Netherlands

Marie Vanier

France

Bernard Vialettes

France

Corinne Vigouroux

France

Lluïsa Vilageliu

Spain

Laura Vilarinho

Portugal

John Vincent

Canada

Jerry Vockley

United States of America

Carsten Wagner

Switzerland

Carina Wallgren-Pettersson

Finland

John Walter

United Kingdom

Ronald Wanders

Netherlands

Ashutosh Wechalekar

United Kingdom

James Weisfeld-Adams

United States of America

Michael West

Canada

Sharon Whatley

United Kingdom

James Whitlock

Canada

Frits Wijburg

Netherlands

Bridget Wilcken

Australia

Ruth Williams

United Kingdom

Steve Wilton

Australia

Uwe Wolfrum

Germany

Pierre Wolkenstein

France

Ed Wraith

United Kingdom

Toshiyuki Yasui

Japan

Terry-Lynn Young

Canada

Giovanna Zambruno

Italy

Christina Zeitz

France

Massimo Zeviani

Italy

Ari Zimran

Israel

Joel Zlotogora

Israel

